# smartSim: simulation of splice aware single cell smart-seq3 data

**DOI:** 10.1093/bioadv/vbaf183

**Published:** 2025-07-30

**Authors:** Marie Van Hecke, Kathleen Marchal

**Affiliations:** IDLab, Department of Information Technology, Ghent University—imec, 9052 Ghent, Belgium; Department of Plant Biotechnology and Bioinformatics, Ghent University, 9052 Ghent, Belgium; Cancer Research Institute Ghent, 9000 Ghent, Belgium; IDLab, Department of Information Technology, Ghent University—imec, 9052 Ghent, Belgium; Department of Plant Biotechnology and Bioinformatics, Ghent University, 9052 Ghent, Belgium; Cancer Research Institute Ghent, 9000 Ghent, Belgium

## Abstract

**Motivation:**

Smart-seq3 is a powerful full-length single-cell RNA sequencing protocol that enables transcript-level quantification and splicing analysis by preserving unique molecular identifier (UMI) information. However, benchmarking computational tools for isoform reconstruction and splicing quantification remains challenging due to the lack of ground truth datasets. Herein, we present smartSim, a Smart-seq3 read simulator designed to generate realistic sequencing data that accurately reflects the complexities of single-cell transcriptomics.

**Results:**

smartSim simulates known and novel splicing events, generates both UMI-containing and internal reads, and mimics protocol-specific biases by leveraging empirical data distributions. Our results show that smartSim-generated data closely resembles real Smart-seq3 datasets in terms of fragment length distributions, internal read counts, and read quality scores. It generates raw sequencing reads in FASTQ format, making it compatible with both genome- and transcriptome-based alignment tools. By extending simulation beyond gene-level quantification, smartSim provides a crucial resource for evaluating and improving computational methods for alternative splicing detection and isoform reconstruction in single-cell RNA sequencing.

**Availability and implementation:**

smartSim is available at https://github.com/MarchalLab/smartSim

## Introduction

Advances in single-cell RNA sequencing (scRNA-seq) have transformed transcriptomics by enabling analysis at single cell resolution. Among the various scRNA-seq protocols, Smart-seq3 (Hagemann-Jensen *et al.* 2022) and Smart-seq3xpress (Hagemann-Jensen *et al.* 2020) stand out for their ability to capture full-length transcripts while incorporating unique molecular identifier (UMI) information, unlike droplet-based methods such as 10X Genomics that show strong 3′ or 5′ bias. These features make Smart-seq3 data particularly valuable for detecting and quantifying splicing events, performing differential splicing analysis, and full-length isoform reconstruction.

Despite its advantages, a key challenge in Smart-seq3-based splicing and isoform reconstruction is the absence of ground truth data for benchmarking computational tools. Existing read simulators such as scReadSim (Yan *et al.* 2023), Splatter (Zappia *et al.* 2017) and muscat (Crowell *et al.* 2020) primarily simulate 10X genomics datasets, which are not suitable for splicing analysis due to their strong 3′ bias. Additionally, Splatter and Muscat only generate gene count matrices, which are useful for evaluating gene-level quantification but insufficient for assessing splice-aware methods. To rigorously assess isoform reconstruction and quantification methods, there is a need for a realistic Smart-seq3 read simulator that mimics the intricate complexities of single-cell transcriptomics. Such a simulator should be capable of simulating reads for isoforms with known and novel splicing events, generating UMI-containing reads as well as internal reads, and mimicking biases introduced by the Smart-seq3 library preparation protocol. Data characteristics such as fragment length and the ratio of internal to UMI-containing reads, which depend on tagmentation time and other protocol specifics, should be faithfully simulated using parameters derived from real datasets.

Here, we introduce smartSim, a novel Smart-seq3 read simulator designed to generate realistic sequencing reads for a single-cell experiment. smartSim is highly versatile, allowing users to simulate data with high or low splicing complexity, different isoform expression profiles, and containing annotated and/or novel transcripts. This flexibility allows researchers to systematically assess how well splicing analysis tools perform under different biologically plausible scenarios. Additionally, it takes a data-driven approach by learning biases from real datasets, such as fragment length distribution, the proportion of internal versus UMI-containing reads, and read qualities. By incorporating these experimentally derived characteristics, smartSim generates realistic Smart-seq3-like data, producing raw FASTQ files.

Because smartSim provides simulation beyond gene-level count matrices, it is compatible with both genome- and transcriptome-based alignment, making it a flexible tool for benchmarking tools that aim to reconstruct isoforms and quantify alternative splicing events in scRNA-seq data. Additionally, it allows to test the impact of various experimental factors and data characteristics, such as read length, fragment length, number of PCR cycles, and single nuclei vs. single cell RNA sequencing. Therefore, smartSim can be used to fine-tune computational methods to match the characteristics of a specific dataset. By generating simulated reads that closely resemble real sequencing data, users can optimize model parameters, adjust filtering thresholds, or evaluate the robustness of their method under different experimental conditions.

## Simulator

### Extracting data characteristics

To generate simulated reads that closely resemble real Smart-seq3 data, we extract key data characteristics from an existing Smart-seq3 aligned dataset. The user can provide this dataset either as a single BAM file or as multiple demultiplexed BAM files, with cell barcode information stored in the “BC” tag and UMI information in the “UB” tag, as commonly obtained from tools such as zUMIs (Parekh *et al.* 2018). It is crucial that the example dataset from which the key features are extracted closely reflects the experimental conditions and protocols of the real data that will eventually be used for analysis.

There are several protocol-specific factors that can significantly influence data characteristics. For example, tagmentation time directly affects fragment lengths, with longer tagmentation times resulting in shorter fragments. Another important factor is whether the input material comes from single nuclei or whole cells. Compared to single-cell RNA-seq, single nuclei data typically exhibits lower read depth and a higher proportion of unspliced RNA fragments, both of which should be reflected in simulations to maintain representativeness.

We provide the function *characterizeData* to extract the following characteristics from a user-provided dataset: the fragment length for UMI-containing and internal reads, the number of reads per UMI, the ratio of internal reads to UMI-containing reads, the fraction of unspliced RNA, and the positional distribution of quality scores across both the read and barcode sequences.

To ensure reliable fragment length estimation without interference from alternative splicing, we focus on a carefully selected subset of genes for parameter estimation. Specifically, we include genes located on chromosomes 1-22 or X/Y that do not overlap with other genes and have only one annotated transcript. By restricting our analysis to these genes, we minimize ambiguity from reads that could otherwise map to multiple overlapping genes. Additionally, by excluding genes with alternative splicing, we ensure that fragment lengths are measured exclusively within exonic regions. For each paired-end alignment mapping to these selected genes, we define the fragment length as the number of exonic positions between the start and end of the alignment. While some of these genes may contain novel splice events, these events are expected to be rare and should have only a minimal effect on the resulting parameter distributions. This approach enables us to extract unbiased fragment length distributions that reflect the sequencing protocol rather than transcript isoform variability.

The extracted characterizations can later be used to ensure the simulated data closely resembles the user-provided dataset. Alternatively, we provide an example Smart-seq3 dataset and the corresponding data characteristics to allow simulation without a user-provided dataset. These data-provided characteristics can be adapted where needed in order to simulate datasets with different experimental conditions.

### Preparing user-defined ground truth

To simulate reads, smartSim requires the sequence of each transcript to simulate and the associated UMI count matrix. While users can define these inputs manually, we also provide options to generate transcript sequences and UMI counts based on a reference genome annotation.

We offer the function *getTranscripts*, which allows users to select *k* random genes, each with *n* annotated transcripts. Alternatively, users can specify a predefined list of genes, from which *n* random annotated transcripts per gene are selected. Genes with fewer than *n* transcripts are excluded.

To allow the introduction of novel splicing events, we provide the functions *addNewJunctions* and *addNewExons*. The *addNewJunctions* function creates novel RNA transcripts by adding new splice junctions that connect existing exons in the reference genome, while *addNewExons* incorporates additional exons into a gene. When adding a novel exon, two additional junctions are inherently introduced, as the exon must be connected to existing exons within the gene. To ensure biologically plausible splicing, we restrict the placement of novel exons such that the newly formed junctions form canonical splice sites, maintaining an AG acceptor and GT donor sequence at both junctions. Users can opt for non-canonical splice sites by using the parameter *canonical = FALSE*.

In addition to transcript sequences, smartSim requires a UMI count matrix for read simulation. We assume an experimental setup with *p* cell populations, each containing *c* cells, though users can modify this configuration to investigate factors such as the impact of rare cell populations.

To generate a transcript-specific UMI count matrix, we follow a three-step approach:

1. Generating the gene UMI count matrix: For each cell population, we generate a gene-level UMI count matrix by first sampling *k* times from the distribution of UMI counts per gene, as extracted in the data characteristics, ensuring that each gene has a minimum count of *n* UMIs. This provides an initial estimate of gene expression levels within each cell population.
  To introduce realistic variability between individual cells within the same cell population, we apply a cell size factor *s*, which accounts for differences in total RNA content across cells. For each individual cell, this factor is randomly sampled from the range $\left[2^{-2},2^{2}\right]$, allowing for variation while preserving overall population-level expression trends. The cell size factor *s* is then used to scale the UMI counts, meaning that each gene’s expression level is adjusted proportionally for each cell, to obtain a gene-level UMI count matrix for each cell.
  By applying this approach, we ensure that expression levels are similar to the user-provided example dataset and that gene expression levels remain biologically plausible, i.e. consistent across cells within the same cell population, yet with natural variability reflective of differences in cell size or RNA capture efficiency.
  Alternatively, users can define custom values for the gene UMI count matrix to more closely resemble the intended data, e.g. generating data with more read coverage.
2. Generating the transcript Percent Spliced In (PSI) matrix: For each gene, we assign transcript-level abundances by randomly distributing the total PSI value of 100% across all transcripts of that gene, reflecting biologically plausible isoform variation. Because splicing is a cell type-specific process, the PSI distribution is expected to be the same for all cells within the same cell population; therefore, we report the transcript-level PSI values per cell population.
  Users have control over how many genes exhibit fixed splicing patterns versus differential splicing across cell populations. For genes with fixed splicing, multiple isoforms of the same gene can be present within a single cell, but the same PSI values are maintained across all cell populations. In contrast, for differentially spliced genes, PSI values are randomly assigned independently for each cell population, simulating variations in splicing regulation between distinct cell populations. This approach provides a controlled setting to assess whether downstream computational methods can not only infer the presence of splice variants but also reliably quantify differential splicing between different cell populations.
3. Generating the unspliced transcript matrix: For each cell population, we generate a gene-level matrix representing the fraction of unspliced transcripts. This is estimated using the number of intronic bases covered relative to the total number of bases covered per gene per cell in the input Smart-seq3 dataset.
  Since splicing and gene expression dynamics are cell type–specific, we assume that the unspliced RNA fraction is generally conserved within a given cell population. Therefore, we report unspliced transcript fractions at the population level rather than per individual cell. Similar to the PSI-value generation process, users can control how many genes share a fixed versus varying unspliced fraction across populations. For genes with fixed splicing, we randomly sample a value from the distribution of unspliced RNA fractions derived from the example data and use the same value across all populations. For the remaining genes, we independently sample values for each gene-cell population pair.
  However, in typical single-cell Smart-seq3 datasets, most genes contain only spliced transcripts, leading to many zero values, even for genes simulated to have potentially varying unspliced fraction across populations. Users specifically interested in modeling differential fractions of unspliced RNA across populations may optionally provide a custom unspliced transcript matrix instead of relying on one generated from the example data.
  Alternatively, to simulate single nuclei data, the user can provide a custom matrix with higher unspliced transcript fractions to better reflect the characteristics of such datasets, allowing users to fine-tune the simulation to their specific experimental conditions.

Finally, the gene UMI count matrix, the transcript PSI matrix, and the unspliced transcript matrix are combined to obtain the transcript UMI count matrix containing the number of unique UMIs to simulate for each (spliced and unspliced) transcript in each cell.

### Smart-seq3 read simulation

We simulate sequencing reads using the input UMI-transcript count matrix and FASTA sequences of each transcript, following steps that closely mimic the Smart-seq3 protocol.

1. UMI and cell barcode assignment: First, we generate a random barcode for each cell. For every UMI entry in the UMI count matrix for that cell, we assign a random UMI sequence. Each transcript is then duplicated according to its UMI count to reflect the transcript abundance. To each of the resulting duplicated transcripts, we append the Smart-seq3 tag (ATTGCGCAATG), the unique random UMI, and GGG, mimicking the Tn5 tagmentation step in Smart-seq3 library preparation.
2. PCR amplification: Next, we simulate PCR amplification, where each tagged transcript undergoes nPCR cycles. To determine a realistic nPCR value similar to the example dataset, we sample from the distribution of the number of reads per UMI observed in real data, as this provides a reliable estimate of the amplification process. However, not all amplified Smart-seq3 fragments are ultimately sequenced. To account for this, we introduce a PCR amplification factor (PAF), which temporarily inflates the number of amplified fragments. This inflation is later corrected during the subsampling step, ensuring a realistic representation of the sequencing process.
3. Generating UMI-containing fragments: We generate UMI-containing reads by extracting fragments from the beginning (5′ end) of each tagged transcript. The fragment lengths are sampled from the distribution of UMI-containing fragment lengths observed in real data. We remove the UMI-containing fragments from the transcript sequences and use the remaining portion of each transcript to generate internal reads.
4. Generating internal fragments: The number of internal reads per transcript is determined based on the empirical distribution of the ratio of internal reads to UMI-containing reads per gene. Specifically, for each transcript, we sample from this distribution and multiply the obtained values by the number of UMI-containing reads for that transcript. We then extract random subfragments from the remaining portion of the transcript, with fragment lengths sampled from the observed distribution of fragment lengths of internal reads in real Smart-seq3 data.
5. Read generation: Using the selected fragments, we simulate paired-end reads by selecting the first 150 bp and the last 150 bp subsequences of each fragment. In Smart-seq3 data, paired-end UMI-containing reads always include the UMI and the 5′ end of the transcript in read 1, along with a downstream portion of the transcript in read 2, thereby preserving the transcript’s strandedness. In contrast, internal reads do not retain strand information. To reflect this, we randomly reverse-complement internal fragments with a probability of 0.5 before extracting read 1 and read 2.
6. Error simulation: To reflect the sequencing noise observed in Smart-seq3 data, we introduce random point mutations. For each base, we introduce a substitution with probability *P*. The error rate *P* is a user-defined variable with default *P* = .005. When an error occurs, the original base is randomly changed to one of the other three nucleotides with equal probability (e.g., an A can be replaced with C, G, or T, each with equal probability).
7. Subsampling: To correct for the PAF adjustment applied earlier, we subsample the final read set to restore the original read counts.
8. Read quality simulation: Finally, we simulate read quality scores by sampling from the empirical distribution of per-position quality scores in real Smart-seq3 data. The resulting reads and their corresponding barcodes, along with their quality scores, are then written to FASTQ files.

## Results

We provide an example dataset derived from E-MTAB-8735 (Hagemann-Jensen *et al.* 2020). This dataset consists of 192 HEK293FT cells that underwent Smart-seq3 library preparation.

To process the data, we assigned cell barcodes and UMIs and aligned reads using zUMIs (Parekh *et al.* 2018) and selected only the reads mapping to chromosome 1 to reduce computational complexity. This example dataset is included with the R package, along with the script used to preprocess the data and a tutorial demonstrating how to simulate reads based on ground truth isoform abundances.

We used this example dataset to evaluate whether the simulator, when provided with this example dataset, could accurately extract the key data characteristics and reproduce a dataset with similar characteristics. We evaluate the simulation protocol by generating reads for 500 genes from the chromosome 1 reference annotation, selecting only genes with at least two transcripts. We simulated two cell populations, each consisting of 100 cells. Note that we do not aim to replicate the full dataset but rather simulate reads for a limited subset of genes.

**Figure 1.**
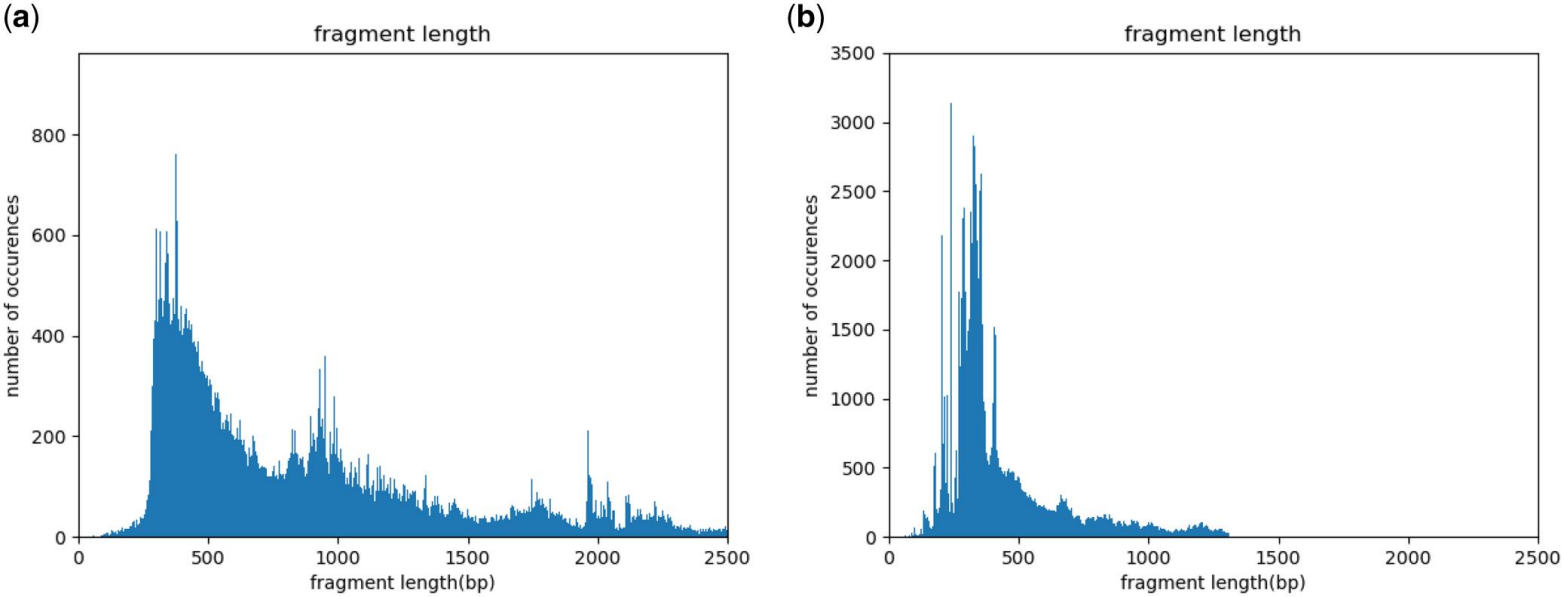
Fragment length for UMI-containing reads, for (a) real data and (b) simulated data.

**Figure 2.**
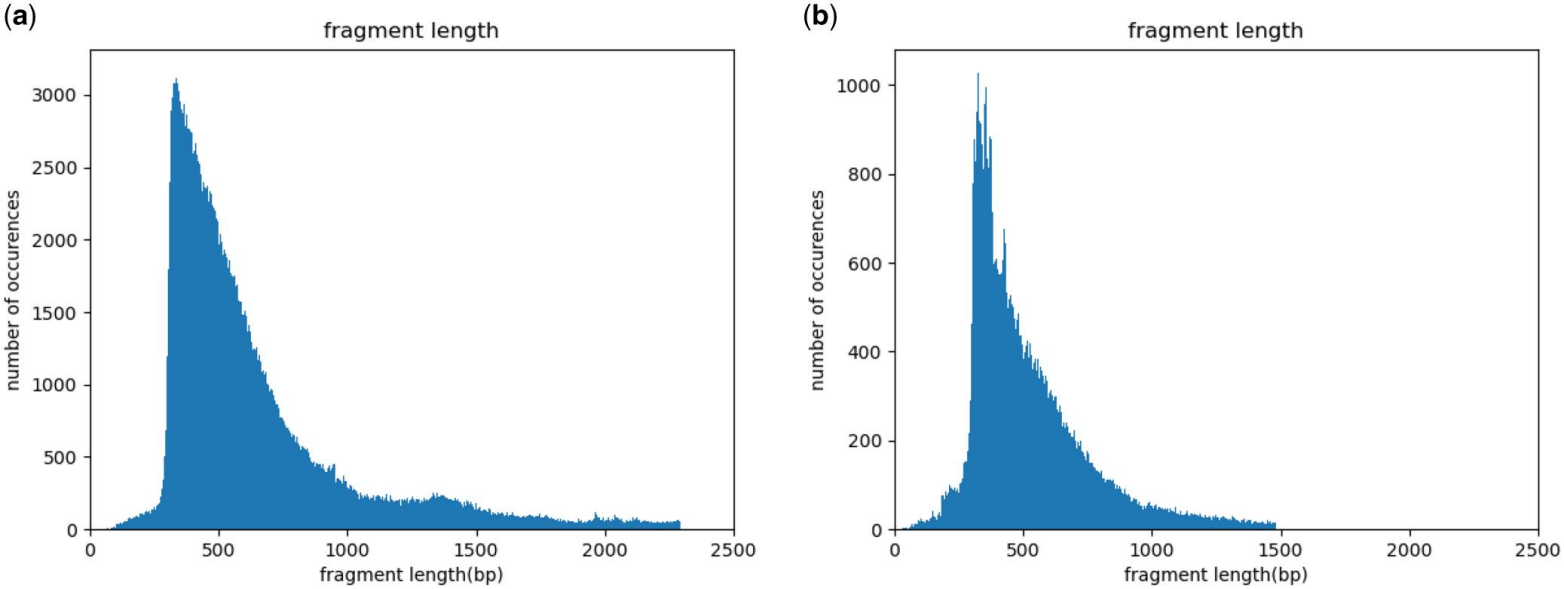
Fragment length for internal reads, for (a) real data and (b) simulated data.

**Figure 3.**
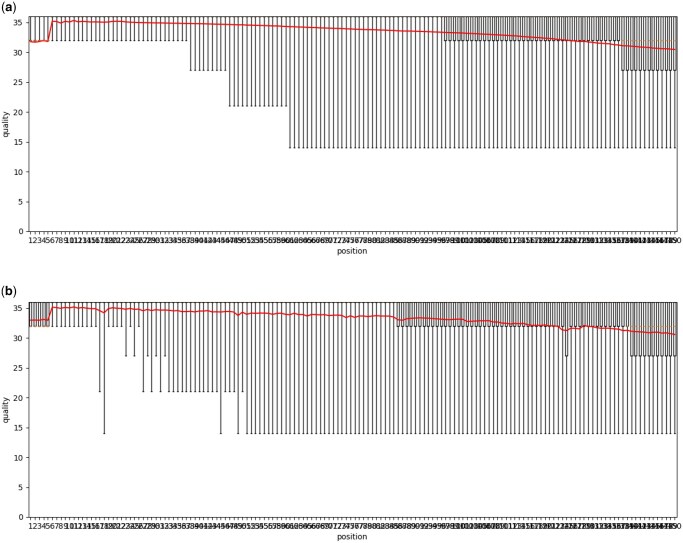
Read quality per position in the read for (a) real data and (b) simulated data.

**Figure 4.**
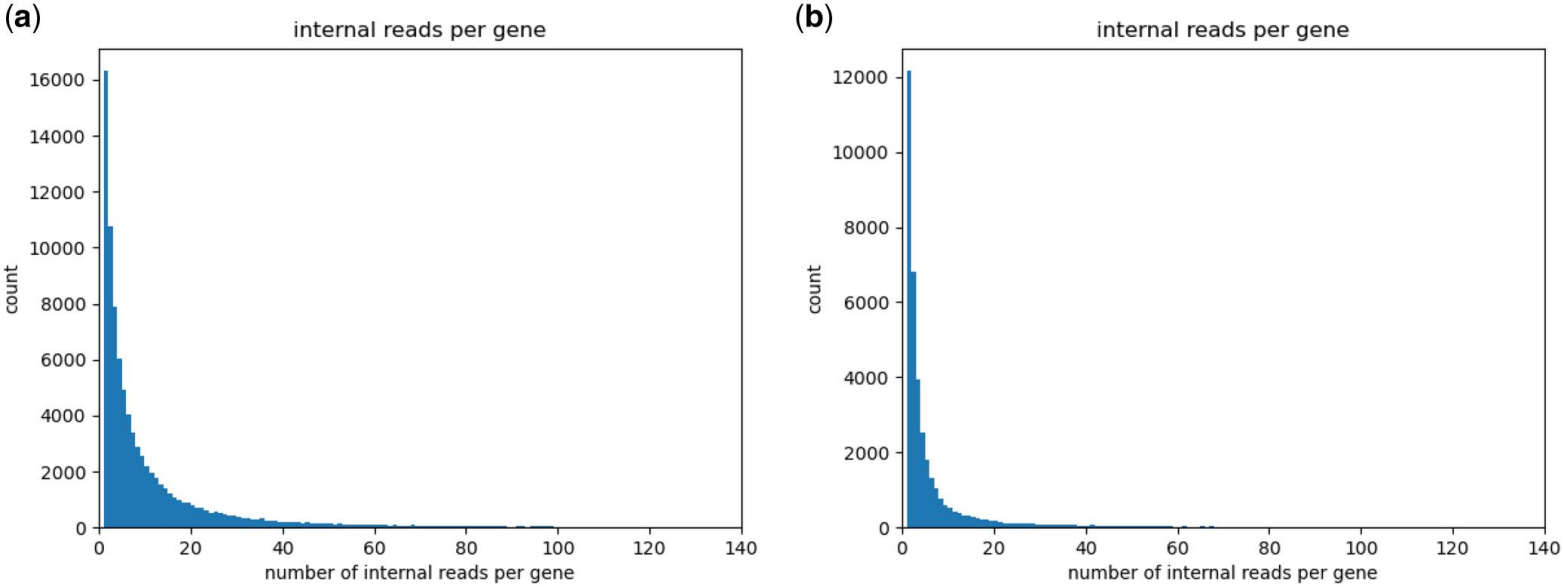
Number of internal reads per gene for (a) real data and (b) simulated data.

**Figure 5.**
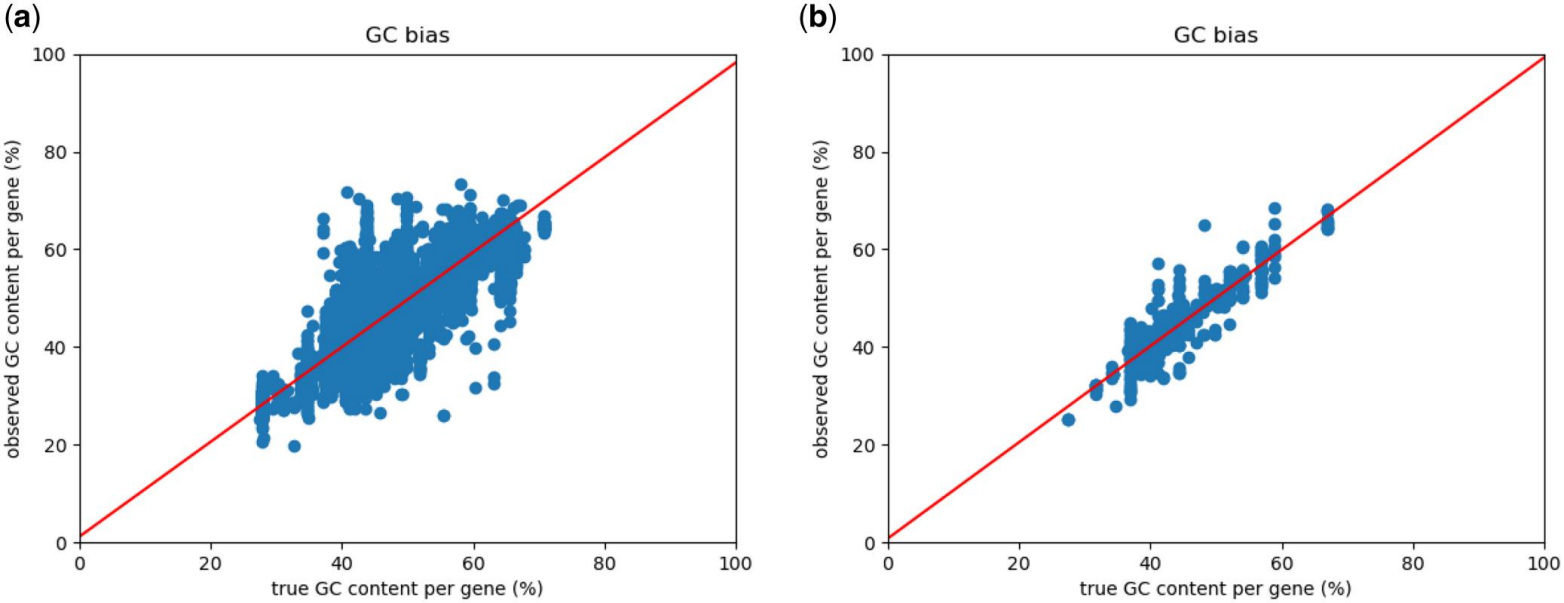
Average GC content of reads versus the GC content of the read itself. Neither real (a) or simulated data (b) show GC bias.

To assess how closely our simulated data matches the provided example Smart-seq3 dataset, we compared the following data characteristics between the example and simulated data: fragment length distributions (Figs. 1 and 2), positional read quality (Fig. 3), the number of internal reads per gene (Fig. 4), and the GC content (Fig. 5).

Since we simulated reads from genes with multiple isoforms, we did not apply the same gene filtering criteria used during parameter estimation, meaning that observed fragment lengths may be influenced by alternative splicing. However, since this bias is present in both the real and simulated datasets, they remain directly comparable. We indeed observe that the distribution of fragment lengths for both UMI-containing (Fig. 1) and internal reads (Fig. 2) is similar between the example and simulated datasets.

Similarly, we observe a strong resemblance between example and simulated data regarding the read quality scores (Fig. 3) and the number of internal reads per gene (Fig. 4).

In addition to the characteristics directly incorporated into the simulation process, we also examined the GC bias (Fig. 5). Because we did not observe GC bias in the Smart-seq3 dataset from Hagemann-Jensen *et al.* (2020), we did not explicitly model it during simulation. As expected, Fig. 5 confirms that our simulated data also lacks GC bias.

To simulate the data described above, smartSim first requires approximately 40 minutes to extract data characteristics from the example dataset, a step that needs to be performed only once per dataset. Generating the simulated data itself takes approximately 28 minutes (1709 ± 114 seconds) and 5.55 ± 0.61 GB of memory. Once completed, the extracted characteristics can be reused for multiple simulations. These benchmarks are based on 10 independent simulation runs on a system with:

- Intel(R) Xeon(R) CPU E5-2698 v3 @ 2.30GHz
- 256 GB RAM
- Ubuntu 22.04.4 LTS
- R version 4.4.1 (platform: x86_64-conda-linux-gnu)

## Conclusion

By extracting empirical data characteristics and protocol-specific biases from the user-provided Smart-seq3 dataset, while offering flexibility to adjust these features to match different target conditions, our simulation framework generates highly realistic Smart-seq3 reads that accurately reflect the complexities of single-cell full-length RNA sequencing. From UMI and barcode assignment to PCR amplification, fragment selection, and sequencing error modeling, each step closely mimics the Smart-seq3 workflow. Additionally, by allowing users to flexibly include both known and novel splicing events, as well as differential splicing across cell populations, our approach provides a robust platform for benchmarking computational methods for isoform reconstruction and alternative splicing quantification. The final output in FASTQ format ensures compatibility with standard alignment and downstream analysis pipelines as well as custom ones, enabling researchers to evaluate and refine their tools under controlled yet realistic experimental conditions.

## Data Availability

The R package, as well as installation instructions and an extensive tutorial with example data, is available on GitHub (https://github.com/MarchalLab/smartSim). smartSim is currently only available for Linux, due to its dependency on the HTSeq package, which is not available for Windows and shows compatibility issues for macOS.
